# Unraveling the Link between Perceived ESG and Psychological Well-Being: The Moderated Mediating Roles of Job Meaningfulness and Pay Satisfaction

**DOI:** 10.3390/bs14070606

**Published:** 2024-07-17

**Authors:** Woo-Sung Choi, Wenxian Wang, Hee Jin Kim, Jiman Lee, Seung-Wan Kang

**Affiliations:** 1 College of Business, Gachon University, Seongnam 13120, Republic of Korea; wschoi2@gachon.ac.kr (W.-S.C.); heejinkim@gachon.ac.kr (H.J.K.); 2 School of Innovation and Entrepreneurship, Wannan Medical College, Wuhu 241002, China; ww@wnmc.edu.cn; 3 School of Business, Yonsei University, Seoul 03722, Republic of Korea

**Keywords:** perceived ESG, psychological well-being, job meaningfulness, pay satisfaction, conservation of resources theory

## Abstract

Enhancing corporate accountability in areas such as environment, social, and governance (ESG) has solidified its role in the discussion on improving corporate resilience and growth. ESG management activities not only augment corporate sustainability and risk control but also influence the professional roles and personal lives of members through their perceived ESG. Historically, most ESG research has centered on the interrelation of corporate ESG endeavors and outcomes, while studies focusing on the influence of perceived ESG on members have been sparse. In this light, our investigation, rooted in the conservation of resources theory, aimed to delineate the mechanistic link between perceived ESG and members’ psychological well-being. This study employed a stratified random sampling technique and collected data across three waves, each spaced four weeks apart. Our sample comprised 325 Korean employees working in administrative, technical, service, and sales roles. This study recruited 325 Korean employees across three time-lagged phases and found that ESG comprehension enhances job meaningfulness, subsequently amplifying psychological wellness. Intriguingly, as pay satisfaction escalates, the mediating role of job meaningfulness between perceived ESG and well-being intensifies. Our study underscores that for organizations to harness perceived ESG to boost psychological well-being via job meaningfulness, managing pay satisfaction is imperative. These findings highlight a crucial policy implication: policymakers must actively promote ESG awareness and incorporate it into employee compensation strategies. This integration is essential to cultivating a healthier, more engaged workforce and driving long-term organizational success.

## 1. Introduction

In the contemporary global landscape, environment, social, and governance (ESG) consideration stands out as a paramount topic of discussion [1,2]. Introduced by the “UN GLOBAL COMPACT”, ESG is an acronym encompassing environmental (E), social (S), and governance (G) aspects, signifying the non-financial elements of a company. Empirical studies have substantiated a positive correlation between ESG and corporate financial outcomes [3,4]. Notably, companies with a higher ESG ranking demonstrate augmented management performance, efficiency, and overall corporate value [5]. Responding effectively to ESG is not a concession to shareholders’ benefits but can actually augment corporate value. Corporate ESG initiatives can positively influence various stakeholders including organizational members, consumers, and investors. Through the lens of the conservation of resources (COR) theory, it is recognized that ESG can significantly influence a company’s long-term sustainability and value by minimizing potential adverse impacts on the environment and society while maximizing the efficacy of governance [6].

The ascendancy of ESG is primarily attributed to the increasing influence of non-financial risks on corporate value. Unlike financial data, information concerning non-financial aspects such as ESG is obtainable based on a company’s disclosure practices. ESG-focused assessments serve a crucial role by furnishing vital data to individual and institutional investors, enabling them to make long-term investments in socially responsible entities [7]. However, given that ESG factors are interrelated, jeopardizing even one aspect can imperil sustainable operations [2].

Historically, ESG research has taken a financial perspective, contending that ESG initiatives are intimately associated with aspects such as market dynamics, leadership, ownership characteristics, risk factors, outcomes, and overall value [4]. Empirical evidence suggests that ESG profoundly impacts corporate value and sustainability in the long run, with ESG investments exerting influence on corporate value through multifaceted channels [8,9,10]. Nevertheless, as corporations advance their ESG management practices, transformative shifts in organizational culture, roles, and processes ensue, in turn precipitating changes in job perception, attitudes, and even roles among internal management and staff [11]. While extensive macro-level research has probed the effects of ESG and corporate social responsibility (CSR) activities on financial and non-financial outcomes, there is a dearth of micro-level research exploring their impact on employees [12,13]. Consequently, there is a burgeoning emphasis on studies concerning organizational changes resulting from ESG management and employees’ perceptions [14,15,16]. 

Perceived ESG is employees’ individual evaluations of the company’s ESG activity level and is a subjective perception [11]. Recent research indicates that employees’ perceptions and evaluations of their company’s ESG activities can profoundly impact their job satisfaction and organizational commitment [17]. These findings suggest that ESG initiatives may significantly enhance employees’ psychological well-being, thereby fostering improved organizational performance and sustainability.

Psychological well-being is a concept that focuses on the pursuit of a meaningful life and personal growth [18]. An examination of current well-being research trends underscores a robust correlation between employees’ psychological well-being and organizational success. Psychological well-being is widely acknowledged as a pivotal factor affecting job performance, creativity, and turnover rates among employees [19]. According to the COR theory, employees are highly motivated to preserve and enhance resources they value, with their psychological well-being improving as these resources become more plentiful [20,21]. This theory provides a framework for understanding how corporate ESG activities can be perceived by employees as valuable resources. While the influence of ESG and CSR activities on employees’ attitudes and behaviors has been a growing research area in recent years [22,23], inquiries into attitude and behavioral shifts based on employees’ perceptions remain scant [22]. This study sought to delineate the relationship mechanism between employees’ ESG cognizance and their psychological well-being from the perspective of the COR theory.

This study diverges from existing studies in two ways. While traditional ESG-related research has primarily fixated on its correlation with corporate performance [3], our investigation introduces a novel perspective by exploring the relationship between perceived ESG and individual psychological well-being through the lens of the COR theory. Moreover, by empirically validating the influence of job meaningfulness and pay satisfaction on psychological well-being derived from perceived ESG, this study elucidates the intricate mechanisms underlying these antecedent variables. In this way, our study extends the research field, offering a more profound understanding of how perceived ESG, job meaningfulness, and pay satisfaction impact employees’ psychological states, thereby making a theoretical contribution. Additionally, organizational managers can glean insights from our research to enhance their members’ psychological well-being and bolster the organization’s overall efficiency and productivity.

## 2. Theoretical Background and Hypotheses

### 2.1. Conservation of Resources Theory

According to the COR theory, individuals exert efforts to accumulate as many resources as possible and preserve and maintain these assets [20]. This predisposition can lead to heightened stress, job dissatisfaction, and a profound sense of loss when confronted with potential or actual resource depletion [24]. Consequently, individuals continuously strive to minimize cognitive or tangible resource losses and replenish perceived or actual resource shortages to maintain their resource pool [24]. Resources are broadly defined to include objects, states, and conditions that individuals value. Within this framework, employees’ perceptions of their organization’s ESG activities can be considered valuable resources [21,24].

Such scarcity can not only hinder job performance but also have repercussions for one’s personal life and emotions. Therefore, the relationship between members’ psychological well-being and its antecedents can be interpreted through the lens of the COR theory. That is, resource loss and depletion can adversely impact members’ psychological well-being. Prior research underscores the significance of workplace dynamics and employee well-being through the lens of the COR theory. Empirical evidence has demonstrated that perceived ESG can bolster employees’ self-esteem and commitment [25]. Organizations should thus establish conditions and policies that allow employees to accumulate diverse resources. Our research meticulously examines the mechanism by which members’ perceived ESG, job meaningfulness, and pay satisfaction influence their psychological well-being using the COR theory.

### 2.2. Perceived ESG and Psychological Well-Being

Perceived ESG refers to the extent to which employees recognize a company’s ESG strategies and commitments [11,26]. Employees with a profound understanding of ESG are exposed to an environment conducive to accepting changes and aspire to contribute to corporate value enhancement and sustainable growth [27]. Intrinsic job motivations linked to creativity and innovative behaviors are tied to job outcomes [28,29]. Employee support and proactive attitudes toward ESG management activities directly influence job outcomes [30]. Active ESG initiatives by companies build a favorable reputation for them. Employees’ recognition of their organization’s positive reputation is linked to positive psychological states and attitudes, such as organizational loyalty and job satisfaction [31,32]. When members perceive corporate ESG activities positively, their feelings toward the organization become more favorable, potentially boosting their psychological well-being [33].

Psychological well-being emphasizes the realization of human potential, signifying the extent to which positive psychological functions are exhibited [34,35]. It includes accepting oneself, exhibiting self-determining behaviors, forming trustworthy relationships, selecting and altering environments, and having a clear motivation and purpose in life [36,37]. Employees experiencing high psychological well-being can more effectively tackle diverse job demands and secure psychological and emotional resources that shield them from resource depletion [38]. 

Psychological well-being is defined as having positive relationships with others, pride and capability in life, and a sense of purpose [39,40]. In work and organizational contexts, job resources improve employees’ psychological well-being, and heightened positive emotions increase their well-being in the work environment [41]. Since psychological well-being is an individual’s perceived positive emotions, it manifests as a positive response to one’s environment and as an outcome of interactions between environmental factors such as policies, supervisors, and colleagues [39,42]. Hence, as employees’ awareness of their company’s ESG management activities rises, this study can anticipate an increase in their psychological well-being.

**Hypothesis** **1:** *Perceived ESG has a positive relationship with psychological well-being. In other words, perceived ESG enhances employees’ psychological well-being.*

### 2.3. Mediating Role of Job Meaningfulness

Job meaningfulness refers to the feeling individuals receive when they believe their role aligns well with their ideals and value system, making them perceive their job as valuable [43,44]. Individuals experience this when they perceive their role as meaningful and beneficial [45]. Such feelings foster motivation toward their work and enhance their attachment to it. Nowadays, many organizational members feel pride and connection when they perceive their organization’s activities as not just profit-driven but also as playing a valuable societal role. Therefore, when ESG activities that align with the company’s vision and objectives are positively recognized by stakeholders, it can amplify members’ sense of job meaningfulness.

When work is seen as meaningful, individuals not only experience self-realization through it but also view it as a medium for personal growth. If one can find significance in their job, it can be a motivational trigger and a springboard for personal development. Experiencing a strong sense of job meaningfulness paves the way for personal growth and boosts work motivation [46]. Those who perceive a high degree of this tend to engage more in organizational citizenship behaviors and display greater resilience.

Those who accumulate a significant sense of job meaningfulness tend to be more focused on their tasks [46]. They actively overcome obstacles in their work and proactively seek new learning opportunities, which increases their potential for growth in their roles. The more individuals find meaning in their jobs, the more likely they are to experience a sense of growth and happiness at work, which can boost everyday well-being. Considering this, job meaningfulness can increase dedication and passion toward one’s role. This passion can, in turn, intensify the sensation of growth, contributing to an individual’s psychological well-being.

**Hypothesis** **2:** *Job meaningfulness acts as a positive mediator between perceived ESG and psychological well-being. Specifically, perceived ESG enhances job meaningfulness, which in turn amplifies employees’ psychological well-being.*

### 2.4. Moderating Effect of Pay Satisfaction and Its Moderated Mediation

From the perspective of the COR theory, financial resources are essential for preserving other resources and mitigating stress [24]. Individuals strive to acquire and maintain resources, and the threat of resource loss can lead to significant stress. Employees who are satisfied with their pay experience less financial stress, enabling them to better appreciate and find meaning in their organization’s ESG activities [47]. Importantly, employees’ perceptions of compensation enhance intrinsic motivation and job meaningfulness. Furthermore, perceptions of compensation, such as pay satisfaction, act as motivational tools, increasing job satisfaction and promoting positive behaviors [48]. As a crucial financial resource, pay satisfaction can enhance the positive impact of perceived ESG on job meaningfulness.

Pay satisfaction reduces financial stress and increases job security, influencing psychological well-being [49]. Intrinsic motivation and monetary rewards interact in complex ways, acting as substitutes in some contexts and complements in others. When employees are satisfied with their pay, they are more likely to find greater meaning in their roles, strengthening the positive relationship between perceived ESG and job meaningfulness. Therefore, pay satisfaction is expected to serve as a moderator in the relationship between employees’ perceived ESG and job meaningfulness. Moreover, through the lens of COR theory, they can understand the mediating role of job meaningfulness in the relationship between perceived ESG and psychological well-being. Job meaningfulness is a significant predictor of eudaimonic well-being, which encompasses aspects of psychological well-being such as self-acceptance, personal growth, and life purpose [50]. Employees who perceive their work as meaningful are more likely to experience higher levels of psychological well-being. Employees who are satisfied with their pay feel valued and appreciated by their organization, which increases intrinsic motivation and job satisfaction. This, in turn, fosters a supportive and fulfilling work environment, enhancing psychological well-being.

Perceptions of income changes significantly impact mental health and well-being, while perceptions of economic uncertainty diminish psychological well-being [51]. Financial resources play a crucial role in this process, helping employees preserve resources, reduce stress, and enhance psychological well-being [52,53]. Therefore, pay satisfaction is predicted to moderate not only the direct relationship between perceived ESG and job meaningfulness but also the indirect relationship between job meaningfulness to psychological well-being. This moderated mediation model suggests that the strength of job meaningfulness’s mediating effect varies with the level of pay satisfaction. Employees satisfied with their pay are more likely to find meaning in their work, thereby amplifying the effect of perceived ESG on psychological well-being through job meaningfulness. Thus, the following hypotheses are proposed:

**Hypothesis** **3:** *Pay satisfaction positively moderates the relationship between perceived ESG and job meaningfulness. Specifically, as the level of pay satisfaction increases, the impact of perceived ESG on job meaningfulness grows stronger.*

**Hypothesis** **4:** *The mediating effect of job meaningfulness between perceived ESG and psychological well-being varies depending on the level of pay satisfaction, moderating the mediation in a positive direction. That is, as pay satisfaction increases, the mediating effect of job meaningfulness, bridging perceived ESG to psychological well-being, intensifies.*

**Figure 1:**
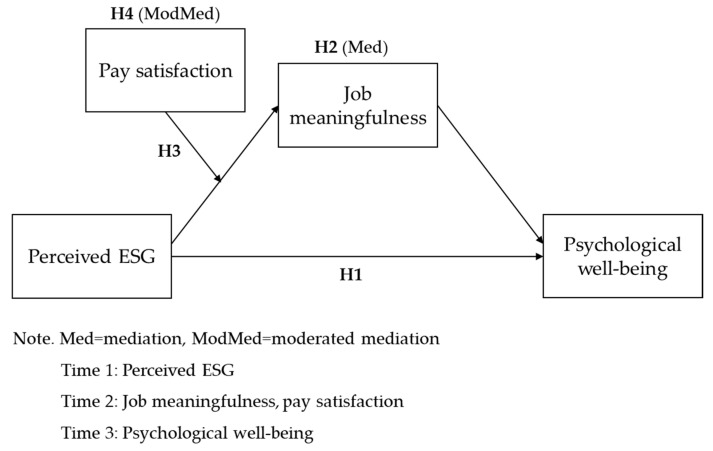
Research model.

Figure 1 illustrates the research model of this study.

## 3. Methods

### 3.1. Sample Collection Procedure and Characteristics

To minimize potential biases resulting from cross-sectional surveys [45,46], this study divided the variables and conducted the investigation in three phases with a one-month gap between them [54]. This study employed the online survey services of the largest panel provider in South Korea and a subsidiary of a renowned global marketing research group, recognized for its extensive and reliable data collection capabilities. By leveraging the services, this study aimed to gather high-quality and dependable data, thereby striving to ensure the validity and integrity of our research findings. The participants were selected using a stratified random sampling method, targeting employees in South Korea who have supervisors in their workplace. 

Before participating in the survey, the researcher informed the respondents about the study’s objectives, procedures, their rights to withdraw participation at any point, and the potential benefits and drawbacks of participation. Following that, they were asked to sign an informed consent form based on the provided information, and data were collected only from those who signed. All collected data were anonymized and securely stored in a separate electronic repository under the supervision of the principal investigator. 

In December 2022, an initial survey was administered via email to 1200 individuals, yielding 672 valid responses after excluding unreliable ones, resulting in a response rate of 56%. In January 2023, a second survey was emailed to the 672 respondents from the initial survey, garnering 450 valid responses and achieving a retention rate of 37.5% relative to the initial 1200 participants. In February 2023, the third survey was sent to the 450 participants who had completed both the first and second surveys, obtaining a total of 325 valid responses after discounting unreliable ones. This represents a retention rate of 27.1% relative to the initial 1200 participants. (See Appendix A for details).

Of the 325 respondents, 70.5% were employed in office positions, 11.7% in production roles, 10.5% in service jobs, and 7.3% in sales or other miscellaneous occupations. The sample consisted of 50.2% females and 49.8% males. Their average age was 41.8 years (SD = 11). Regarding their highest educational qualifications, 53.2% had completed a 4-year bachelor’s degree, 20.9% had a 2-year bachelor’s degree, 18.8% were high school graduates, 5.6% held master’s degrees, and 1.5% had doctorate degrees. The average tenure at their current company was 7.7 years (SD = 6.8). In total, 50.5% of respondents had children, while 49.5% did not.

### 3.2. Measurements

The variables in this study were measured using a five-point Likert scale (1 = Strongly Disagree to 5 = Strongly Agree). The original questionnaire, initially developed in English, was translated into Korean and then reviewed and refined by a bilingual expert. To ensure translation accuracy, the Korean version was subsequently back-translated into English. This back-translation process facilitated the comparison of linguistic and semantic consistency with the original text, thereby affirming the translation’s validity (see Appendix B) [55].

#### 3.2.1. Perceived ESG

Perceived ESG was measured utilizing five items on CSR perception developed by Lichtenstein et al. [56]. Example items are “Our company is committed to using a portion of its profit to help nonprofits”, and “Our company integrates charitable contributions into its business activities”. The Cronbach’s alpha was 0.95.

#### 3.2.2. Pay Satisfaction

Pay satisfaction was gauged using four items developed by Heneman and Schwab [57]. Example items are “I am satisfied with my overall level of pay”, and “I am satisfied with my take-home pay”. The Cronbach’s alpha was 0.97.

#### 3.2.3. Job Meaningfulness

Job meaningfulness was measured using three items developed by Spreitzer [43]. Example items are “The work I do is very important to me”, and “My job activities are personally meaningful to me”. The Cronbach’s alpha was 0.89.

#### 3.2.4. Psychological Well-Being

Psychological well-being was assessed with 6 items using the purpose in life dimension (3 items) and the personal growth dimension (3 items) from psychological well-being scale developed by Ryff and Davidson [36], following Waterman’s proposition [58]. Example items are “Some people wander aimlessly through life, but I am not one of them”, and “For me, life has been a continuous process of learning, changing, and growth”. The Cronbach’s alpha for all items was 0.90, the Cronbach’s alpha for the purpose in life dimension was 0.85, and the Cronbach’s alpha for the personal growth dimension was 0.87.

#### 3.2.5. Control Variables

To ascertain the relationships between the variables presented in our research model with more clarity, this study referred to preceding studies that dealt with similar research variables. Accordingly, this research employed age, gender, education level, tenure, and child status as control variables [59,60,61].

### 3.3. Common Method Bias

To mitigate the likelihood of common method bias, the survey was executed in three staggered phases. For the sample data (n = 325) that were the subject of analysis in this paper, the results from Harman’s single-factor test indicated that the proportion of the first factor was 37.04%. This suggests that our research data did not suffer from significant common method bias [62].

### 3.4. Analytical Strategy

To validate the legitimacy of the research model, confirmatory factor analysis (CFA) was carried out. For testing our research hypotheses using hierarchical regression analysis, STATA 17.0 (Stata Corp., College Station, TX, USA) was employed. Analyses to verify the mediated effect and modulated mediated effect utilizing bootstrapping followed the recommendations of Hayes [63].

## 4. Results

### 4.1. Descriptive Statistics and Correlations

Table 1 presents the mean, standard deviation, correlation, and Cronbach’s alpha values. Significant correlations consistent with the hypotheses were observed among the research variables.

### 4.2. Confirmatory Factor Analysis

Table 2 presents the outcomes of the confirmatory factor analysis (CFA) performed to assess the construct validity of the research variables. The chi-square to the degree of freedom ratio was 1.55, which is well below the acceptable threshold of 3.00. Additionally, the comparative fit index (CFI) and Tucker–Lewis index (TLI) were 0.979 and 0.974, respectively, both exceeding the preferred benchmark of 0.950 [64]. The root mean square error of approximation (RMSEA) stood at 0.041, well below both the maximum allowable limit of 0.080 and the desired standard of 0.050 [64]. Based on these fit indices, the hypothesized four-factor model exhibited excellent fit. After comparing it with three alternative models, the four-factor structure was determined to be the most fitting. All variables met the standard criteria with average variance extracted (AVE) values exceeding 0.5 and composite reliability values surpassing 0.7. The correlation coefficients between variables were found to be below the square root of the AVE [64]. Moreover, all standardized factor loadings of the predictor variables were greater than 0.50 [64].

This study assessed the normality of our data by examining the skewness and kurtosis values of all measured variables. The skewness values ranged from −0.70 to 0.18, well within the stringent threshold of an absolute value of 2, and the kurtosis values ranged from 2.17 to 3.27, also within the stringent threshold of an absolute value of 7 [65]. Thus, this study determined that there are no significant issues with the normality assumption for our data.

### 4.3. Hypothesis Testing

To test Hypotheses 1 and 3, hierarchical multiple regression analyses were employed. In contrast, Hypotheses 2 and 4 were tested using the bootstrapping method [63]. Upon examining Hypothesis 1, as showcased in Model 5 in Table 3, a significant positive relationship was observed between perceived ESG and psychological well-being. Model 5 demonstrated superior explanatory power compared to Model 4, thereby supporting Hypothesis 1.

For Hypothesis 2, an indirect effect analysis using 10,000 bootstrapping iterations was performed. The results, as detailed in Table 4, indicated that the 95% confidence interval (95% CI = [0.02, 0.10]) did not encompass 0, affirming the support for Hypothesis 2. 

Additionally, the Sobel test confirmed the significance of the mediation effect (coefficient = 0.05, *p* < 0.01). The hierarchical multiple regression results in Table 2, according to the causal steps approach, also indicate that a mediation effect is present. Firstly, perceived ESG is significantly related to psychological well-being, with Model 5 showing a coefficient of 0.25 (*p* < 0.001). Secondly, perceived ESG is significantly related to job meaningfulness, as indicated by Model 2, which shows a coefficient of 0.17 (*p* < 0.01). Thirdly, when both perceived ESG and job meaningfulness were included in the analysis, job meaningfulness was significantly related to psychological well-being, as seen in Model 6 with a coefficient of 0.41 (*p* < 0.001). Lastly, the influence of perceived ESG on psychological well-being decreased when job meaningfulness was included in the model. Specifically, the coefficient for perceived ESG in Model 5 was 0.25 (*p* < 0.001), which decreased to 0.18 (*p* < 0.001) in Model 6. These findings suggest the presence of a partial mediation effect.

**Figure 2:**
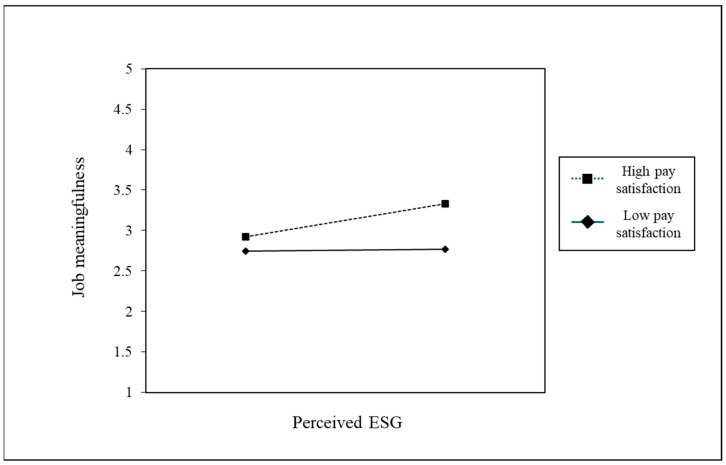
Moderating effect of pay satisfaction level on the relationship between perceived ESG and job meaningfulness.

Hypothesis 3 posited that the relationship between perceived ESG and job meaningfulness is moderated by pay satisfaction. In Model 3 in Table 3, a significant interaction was identified between perceived ESG, pay satisfaction, and job meaningfulness. Model 3 presented a significantly enhanced explanatory capability compared to Model 2. Subsequent simple slope tests revealed that the relationship between perceived ESG and job meaningfulness was significant at a high level (+1 SD) of pay satisfaction but not at a low level (−1 SD) (see Figure 2). Thus, a moderation effect was confirmed [66].

Hypothesis 4 (moderated mediation effect) was tested using a bootstrapped indirect effect test with 10,000 iterations, referencing Hayes [63]. The mediating effect (i.e., the influence of perceived ESG on psychological well-being via job meaningfulness) was examined at high (+1 SD) and low (−1 SD) pay satisfaction levels. As depicted in Table 5, the mediating effect was significant at high pay satisfaction level (with a confidence interval excluding 0; LLCI = 0.02, ULCI = 0.12) but not at low level (confidence interval includes 0; LLCI = −0.03, ULCI = 0.08). Thus, Hypothesis 5 was supported.

**Figure 3:**
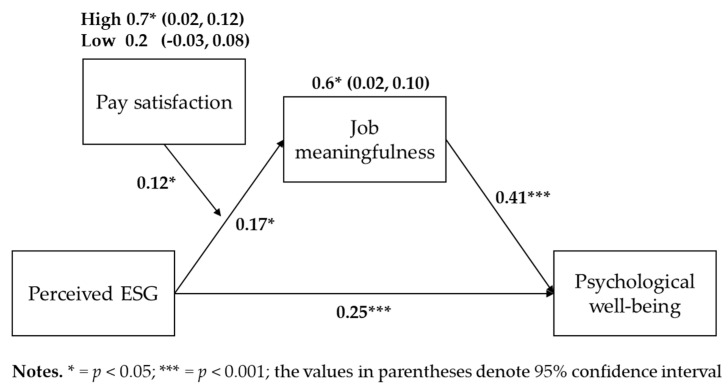
Regression and bootstrapped indirect effect test result.

The verification of hypotheses and the relationships between research variables, established through regression analysis and bootstrapping mediation analysis, are depicted in Figure 3.

## 5. Discussion

### 5.1. Theoretical Contributions

This study contributes to the expansion of the COR theory and clarifies the mechanisms of psychological well-being by presenting several significant insights. ESG is considered an essential component for the survival and growth of modern corporate organizations. When companies pursue ESG management activities, changes in organizational culture, job roles, and processes ensue, leading to shifts in task perception and attitudes, plus alterations among internal employees [11]. While there have been numerous macro-level studies on the impact of ESG activities on a company’s financial and non-financial outcomes, micro-level studies on their influence on employees are relatively sparse [12]. As a result, research highlighting changes due to ESG management and employees’ perceptions is emphasized [14,15]. In this context, our research aimed to elucidate the mechanism between employees’ perceived ESG and their psychological well-being from the COR perspective.

First, the relationships among perceived ESG, job meaningfulness, and pay satisfaction were validated. Following various preceding studies on perceived ESG, this study applied the COR perspective to clarify the structure and relations of these variables. Existing ESG research has predominantly focused on macro-level studies about the impacts of ESG activities on the financial and non-financial performance of companies, leaving a gap in micro-level studies examining their effects on employees [4,12]. This study addressed the need for micro-level investigations on perceived ESG, bridging the research gap and enhancing the theoretical foundation of psychological well-being. This study empirically validates the hypothesis that job meaningfulness and pay satisfaction act as antecedents of psychological well-being, as theorized within the COR framework [21,24]. Furthermore, our research extends the theory by elucidating the relationship and mechanism between perceived ESG and psychological well-being, thereby reinforcing prior findings that analyzed the relationship between job meaningfulness [67], income and pay satisfaction [47,48,51], and psychological well-being through the COR perspective.

Second, this study empirically demonstrated the mediating role of job meaningfulness in the relationship between perceived ESG and psychological well-being. The revelation that job meaningfulness not only influences employees’ attitudes and behaviors at work, such as organizational commitment and organizational citizenship behavior but also impacts their quality of life accentuates the significance and pathways of influence of perceived ESG.

Finally, this study elucidated the mechanism by which ESG awareness, job meaningfulness, and salary satisfaction influence psychological well-being, through the lens of the COR theory. However, alternative theoretical perspectives or a comprehensive approach that explains the relationship between ESG awareness and psychological well-being could be posited. For instance, the moderating effect of salary satisfaction might be explicable from the vantage point of stakeholder theory [68,69], social conflict theory, or agency theory, pertaining to the organizational dilemma of allocating limited resources either for external ESG endeavors or internal employee rewards such as salary [70,71]. Moreover, frameworks such as the two-factor theory could be invoked, suggesting that without fulfilling basic compensation, the satisfaction mechanism based on ESG awareness may not operate [72]. This study identified ESG awareness, salary satisfaction, and job importance as resources that explain individual psychological mechanisms, providing a foundation for integrating various theoretical backgrounds and perspectives in the future.

### 5.2. Practical Implications

This study conveys the following managerial insights. First, by empirically demonstrating that awareness of ESG can act as a precursor to psychological well-being, it provides guidance on what organizational endeavors are required to enhance members’ quality of life. Investment in ESG initiatives may not lead to improved psychological well-being among employees if they remain unaware of the company’s level of ESG engagement. Consequently, it is crucial for managers to consistently gauge employees’ perceived ESG and educate them about the company’s ESG initiatives.

Second, the study sheds light on the role of job meaningfulness as a mediator in the relationship between perceived ESG and psychological well-being, thereby offering a roadmap for boosting psychological well-being. This implies that organizational leaders must regularly communicate and devise structural systems to ensure that each individual can empathize with the value and purpose of their tasks.

Third, while perceived ESG is anticipated to enhance employees’ job meaningfulness, this effect is contingent upon the presence of satisfactory pay. Therefore, it is essential for managers and organizational leaders to ensure that employees receive competitive and fair compensation before promoting the company’s ESG and social contributions. This foundational aspect of employee satisfaction must be addressed to establish a stable ground upon which the benefits of ESG initiatives can be realized. Effective compensation strategies should be regularly reviewed to ensure they meet industry standards and address any existing disparities, thereby reinforcing employees’ trust and loyalty to the organization.

Fourth, our findings reveal that employees’ perceptions of rewards significantly moderate the relationship between perceived ESG and job meaningfulness. The subjective nature of compensation perceptions, including pay satisfaction, is influenced by the organization’s transparency in resource allocation and decision-making processes. It is, therefore, vital for organizations to maintain open lines of communication regarding how resources are allocated to ESG initiatives and to highlight their long-term benefits for both the company and its employees. This transparency fosters a culture of trust and collaboration, aligning employees’ personal values with the organization’s long-term sustainability goals. By effectively communicating the impact of ESG investments, managers can enhance employee commitment and support for the organization’s sustainability agenda.

### 5.3. Research Limitations and Future Research Directions

While this study imparts valuable insights for both scholars and practitioners, some limitations necessitate future augmentation. First, this study’s sampling method and scope present limitations. Despite utilizing data from three staggered surveys, each variable’s measurement was confined to specific timeframes, rendering a cross-sectional study impossible. Future studies should consider a longitudinal research design.

Second, the survey targeted Korean employees, and their perceptions and attitudes might have been influenced by their cultural background, necessitating caution when generalizing the findings to different cultures. Future studies exploring the effect of perceived ESG on psychological well-being in diverse cultural contexts might bring forth novel insights or comparative analyses.

Third, the study’s variable measurements stemmed from identical sources, leading to potential common method bias. Although the confirmatory factor analysis differentiated the variables effectively, subsequent research should bear this issue in mind.

Fourth, this research clarified the mechanism through which perceived ESG, job meaningfulness, and pay satisfaction influence psychological well-being from the COR theory viewpoint. Nevertheless, integrating or suggesting other theoretical frameworks that can elucidate the relationship between perceived ESG and psychological well-being could yield further insight.

Fifth, this study serves as an initial investigation into ESG, traditionally examined as a macro-level variable, approached from a micro-level perspective within organizations [73]. Future research should aim to further expand the scope and depth of this inquiry. Extending the research to include a broader array of organizational contexts and industry sectors would be particularly valuable. By examining diverse organizational environments, researchers can better understand how specific industry practices and standards impact the relationship between perceived ESG and employee well-being. Additionally, future studies could explore the role of individual differences, such as personality traits, values, and career stages, in moderating the effects of perceived ESG on job meaningfulness and psychological well-being. Understanding these individual factors could provide more personalized insights and practical applications for organizational leaders.

Sixth, integrating qualitative methods, such as in-depth interviews and focus groups, with quantitative approaches could enrich the data and provide a more nuanced understanding of how employees perceive and are affected by ESG initiatives [74]. This mixed-methods approach would allow for the exploration of complex and context-specific factors that quantitative methods alone might overlook.

Finally, future research should examine the potential long-term outcomes of perceived ESG on employee well-being and organizational performance. Longitudinal studies tracking these variables over extended periods would provide valuable insights into the sustainability of ESG impacts and their implications for organizational success. This approach would also enable researchers to identify causal relationships and better understand the temporal dynamics of perceived ESG and their effects.

### 5.4. Conclusions

This study sought to decode the “black box” of how perceived ESG impacts employee psychological well-being, particularly concentrating on the mechanism underpinning this influence. According to the COR theory, employees with positive perceptions of their organization’s ESG actions are likely to experience enhanced job meaningfulness, which acts as an organizational resource, consequently amplifying individual psychological well-being. The empirical results also highlighted that pay satisfaction, a representative perception of rewards, positively moderates the effect of perceived ESG on job meaningfulness, enhancing its beneficial influence on psychological well-being through job meaningfulness. 

Employees who are satisfied with their pay experience reduced financial stress, enabling them to better understand and find meaning in their organization’s ESG activities. Notably, employees’ perceptions of compensation enhance intrinsic motivation and job meaningfulness. Furthermore, pay satisfaction helps employees conserve resources, alleviate stress, and improve psychological well-being. This study, while supporting existing research on the antecedents of psychological well-being from the perspective of COR theory, clarifies that pay satisfaction is a necessary condition for perceived ESG to enhance psychological well-being.

This research not only provides empirical evidence of the effect of employees’ perceptions of corporate ESG management activities on their psychological well-being via job meaningfulness but also offers concrete insights into organizational change and human resource management for ESG.

## Figures and Tables

**Table 1:** Means, standard deviations, correlations, and reliabilities.

Variable	Mean	SD	1	2	3	4	5	6	7	8	9
1. Age	41.78	10.97	-								
2. Female	0.50	0.50	−0.09	-							
3. Education	2.50	0.92	−0.14 *	0.03	-						
4. Tenure	7.67	6.84	−0.12 *	0.44 ***	0.01	-					
5. Child	0.49	0.50	−0.13 *	0.47 ***	0.10	0.27 ***	-				
6. Perceived ESG	2.49	0.97	0.03	−0.03	0.19 ***	0.08	0.10	(0.95)			
7. Job meaningfulness	3.40	0.84	0.22 ***	−0.01	0.09	0.08	0.33 ***	0.21 ***	(0.97)		
8. Pay satisfaction	2.40	0.92	0.14 **	−0.10	0.07	0.15 **	0.15 **	0.23 ***	0.29 ***	(0.89)	
9. Psychological well-being	3.38	0.73	0.06	−0.08	0.15 **	−0.01	0.19 ***	0.29 ***	0.47 ***	0.23 ***	(0.90)

Notes. n = 325; * *p* < 0.05, ** *p* < 0.01, *** *p* < 0.001 (two-tailed); the values in parentheses denote Cronbach’s alphas; Age = years; Female, male = 0, female = 1; Education = highest education level achieved: 1 = high school graduate, 2 = 2-year college graduate, 3 = 4-year university graduate, 4 = master’s graduate, 5 = Ph.D. holder; Tenure = organizational tenure (year); Child, no child = 0, has child = 1. Abbreviation: ESG: Environmental, social and governance.

**Table 2:** Confirmatory factor analysis results.

Model	χ^2^(df)	CFI	TLI	RMSEA	Δχ^2^(Δdf)
Research model (4 factor)	309.43(199) ***	0.98	0.97	0.04	
Alternative model 1 (3 factor) ^1^	1957.62(207) ***	0.85	0.82	0.09	1648.19(8) ***
Alternative model 2 (2 factor) ^2^	2581.50(214) ***	0.70	0.65	0.13	2272.07(15) ***
Alternative model 3 (1 factor) ^3^	3580.54(220) ***	0.48	0.42	0.17	3271.11(21) ***

Notes. n = 325; *** *p* < 0.001; ^1^ Three-factor model with perceived ESG and pay satisfaction on the same factor. ^2^ Two-factor model with perceived ESG, pay satisfaction, and job meaningfulness on the same factor. ^3^ One-factor model with perceived ESG, pay satisfaction, job meaningfulness, and psychological well-being on the same factor. Abbreviations: CFI, comparative fit index; TLI, Tucker–Lewis index; RMSEA, root mean square error of approximation.

**Table 3:** Hierarchical multiple regression.

Variable	Job Meaningfulness			Psychological Well-Being		
	Model 1	Model 2	Model 3	Model 4	Model 5	Model 6
Age	0.03	0.04	0.02	−0.08	−0.06	−0.08
Female	0.04	0.04	0.06	−0.05	−0.05	−0.07
Education	0.09	0.06	0.05	0.14 *	0.09	0.07
Tenure	−0.03	−0.04	−0.07	−0.06	−0.08	−0.06
Child	0.33 ***	0.30 ***	0.31 ***	0.26 ***	0.22 **	0.10
Perceived ESG		0.17 **	0.13 *		0.25 ***	0.18 ***
Pay satisfaction			0.22 ***			
ESG × Pay satisfaction			0.12 *			
Job meaningfulness						0.41 ***
R^2^	0.12	0.15	0.21	0.07	0.13	0.27
ΔR^2^		0.03	0.06		0.06	0.14
adj R^2^	0.10	0.13	0.19	0.05	0.11	0.26
F	8.55 ***	9.03 ***	10.23 ***	4.72 ***	7.85 ***	17.01 ***
F_inc_		10.19 **	11.95 ***		21.93 ***	62.85 ***

Notes. n = 325; * = *p* < 0.05; ** = *p* < 0.01; *** = *p* < 0.001 (two-tailed test). The results are standardized regression coefficients.

**Table 4:** Results of bootstrapped indirect effect test.

	Dependent Variable: Psychological Well-Being			
Mediating Variable	Indirect Effect	SE	95% CI	
			LLCI	ULCI
Job meaningfulness	0.06	0.02	0.02	0.1

Notes. n = 325, number of bootstrapping iterations = 10,000. Abbreviations: SE, standard error; CI, confidence interval; LLCI, lower limit of confidence interval; ULCI, upper limit of confidence interval.

**Table 5:** Results of conditional bootstrapped indirect effect test.

		Dependent Variable: Psychological Well-Being			
Moderating Variable	Level of Moderator	Indirect Effect	SE	95% CI	
				LLCI	ULCI
Pay satisfaction	Low (−1 SD)	0.02	0.03	−0.03	0.08
	High (+1 SD)	0.07	0.02	0.02	0.12

Notes. n = 325, number of bootstrapping iterations = 10,000. Abbreviations: SD, standard deviation; SE, standard error; LLCI, lower limit of confidence interval; ULCI, upper limit of confidence interval.

## Data Availability

The raw data supporting the conclusions of this article will be made available by the authors, without undue reservation, to any qualified researcher.

## Appendix B. Measurement

**ESG (α = 0.95) (Lichtenstein, Drumwright, & Braig, 2004)** [56]

1. Our company is committed to using a portion of its profit to help nonprofits.
2. Our company gives back to the communities in which it does business.
3. Local nonprofits benefit from our company’s contributions.
4. Our company integrates charitable contributions into its business activities.
5. Our company is involved in corporate giving.

**Pay satisfaction (α = 0.89) (Heneman & Schwab, 1985)** [57]

1. I am satisfied with my overall level of pay.
2. I am satisfied with my current salary.
3. I am satisfied with my take-home pay.
4. I am satisfied with the size of my current salary.

**Job meaningfulness (α = 0.97) (Spreitzer, 1995)** [43]

1. The work I do is very important to me.
2. My job activities are personally meaningful to me.
3. The work I do is meaningful to me.

**Psychological well-being (α = 0.90) (Ryff & Davidson, 2010)** [36]

**Purpose in life**

1. Some people wander aimlessly through life, but I am not one of them.
2. I live life one day at a time and do not really think about the future. (R)
3. I sometimes feel as if I have done all there is to do in life. (R)

**Personal growth**

4. For me, life has been a continuous process of learning, changing, and growth.
5. I think it is important to have new experiences to challenge how I think about myself and the world.
6. I gave up trying to make big improvements or changes in my life a long time ago. (R)
